# Treatment of Capillary Bronchitis in Children by Warm Vapor

**Published:** 1871-08

**Authors:** 


### Miscellaneous.

### Treatment of Capillary Bronchitis in Children by Warm Vapor.

Prof. Abelin, of Stockholm, observes that capillary bronchitis, with its usual sequelæ, collapse, broncho-pneumonia, and emphysema, belongs to the most dangerous diseases of childhood. It most frequently originates in a simple bronchitis which extends from the larger into the smaller bronchia. It is sometimes, however, a primary affection, and attacks with extreme violence children that are apparently in rude health. When primary, it is characterized not only by the intensity, but by the rapidity of its progress. The symptoms resemble more the direct action of poison than a catarrhal inflammatory affection. From the date of occurrence of the first symptoms the patient passes into a state of collapse, the temperature sinks, dyspnœa and cyanosis augment, and ultimately complete anæsthesia supervenes. The course is usually so rapid that the little patient often succumbs in the course of twenty-four hours, and not seldom in from twelve to twenty-four hours. The usual accompaniments of capillary bronchitis, broncho-pneumonia and emphysema, do not appear in such cases to have sufficient time to develop, or at least they are undiscoverable in the dead body. Death, as in croup, results from the rapid progress of asphyxia. After death, only a quantity of secretion is found accumulated in the bronchia, together with much epithelial *debris*, and more or less congestion of the posterior lobes of the lungs. In capillary bronchitis, and especially in the paralytic form, every kind of debilitating treatment should be avoided. Abelin, in the earlier period of practice, adopted antiphlogistic treatment, and rarely saw a child recover. Subsequently, he prescribed tonics and stimulants, (quinine, musk, camphor, turpentine) with better results; but all these remedies were far surpassed in value by the mode of treatment long employed in his hospital, by the respiration of warm vapor, or rather of placing the patient in a hot air bath. The children were placed in a properly constructed small chamber, in which was a vessel of water that was kept boiling day and night. Here the patient was retained for days and even weeks, until complete recovery, which, however usually soon took place. The result of comparison with other modes of treatment was most satisfactory. The percentage of death, which in 1864, amounted to 48, diminished in 1868 to 18. M. Abelin has also found great benefit from breathing the vapor of hot water in pneumonia. Lobular pneumonia may likewise be thus treated, and here, in addition, turpentine embrocations and cataplasms are to be supplied. In lobular pneumonia M. Abelin first gave calomel, or, if diarrhea be present, small doses of calomel with opium or morphia, inf. ipecacuanhæ, with vin. liquiritiæ, thebaicum, and syrup. scillæ, and as soon as the symptoms give way a turpentine emulsion internally and fly blisters externally.—*Journal fur Kinderkrankheiten.*—*Boston Medical Journal.*

